# Expression of Zinc Transporter Genes in Rice as Influenced by Zinc-Solubilizing *Enterobacter cloacae* Strain ZSB14

**DOI:** 10.3389/fpls.2016.00446

**Published:** 2016-04-06

**Authors:** Selvaraj Krithika, Dananjeyan Balachandar

**Affiliations:** Department of Agricultural Microbiology, Tamil Nadu Agricultural UniversityCoimbatore, India

**Keywords:** metal transporter, rice, zinc solubilizing bacteria, zinc uptake, ZIP genes

## Abstract

Zinc (Zn) deficiency in major food crops has been considered as an important factor affecting the crop production and subsequently the human health. Rice (*Oryza sativa*) is sensitive to Zn deficiency and thereby causes malnutrition to most of the rice-eating Asian populations. Application of zinc solubilizing bacteria (ZSB) could be a sustainable agronomic approach to increase the soil available Zn which can mitigate the yield loss and consequently the nutritional quality of rice. Understanding the molecular interactions between rice and unexplored ZSB is useful for overcoming Zn deficiency problems. In the present study, the role of zinc solubilizing bacterial strain *Enterobacter cloacae* strain ZSB14 on regulation of Zn-regulated transporters and iron (Fe)-regulated transporter-like protein (ZIP) genes in rice under iron sufficient and deficient conditions was assessed by quantitative real-time reverse transcription PCR. The expression patterns of *OsZIP1, OsZIP4*, and *OsZIP5* in root and shoot of rice were altered due to the Zn availability as dictated by Zn sources and ZSB inoculation. Fe sufficiency significantly reduced the root and shoot *OsZIP1* expression, but not the *OsZIP4* and *OsZIP5* levels. Zinc oxide in the growth medium up-regulated all the assessed ZIP genes in root and shoot of rice seedlings. When ZSB was inoculated to rice seedlings grown with insoluble zinc oxide in the growth medium, the expression of root and shoot *OsZIP1, OsZIP4*, and *OsZIP5* was reduced. In the absence of zinc oxide, ZSB inoculation up-regulated *OsZIP1* and *OsZIP5* expressions. Zinc nutrition provided to the rice seedling through ZSB-bound zinc oxide solubilization was comparable to the soluble zinc sulfate application which was evident through the ZIP genes’ expression and the Zn accumulation in root and shoot of rice seedlings. These results demonstrate that ZSB could play a crucial role in zinc fertilization and fortification of rice.

## Introduction

Zinc (Zn) is a critical micronutrient responsible for several cellular functions in plant and its deficiency causes decrease in plant growth and yield. Zn deficiency in major food crops, apart from yield loss, reduced the Zn content of grains and subsequently causes serious problems in human nutrition. Rice, the staple diet for more than 560 million people of world, is one of the “most sensitive" crops for Zn nutrition. Nearly 50% of the rice-growing soils are under Zn-deficient and rice grown on these soils generally produce low yield with poor nutritional quality. For incidence, Zn-sufficient rice had about 40 mg/kg of Zn in its grains, whereas Zn-deficient rice accumulated less than 10 mg/kg of grain-Zn (Wissuwa et al., 2008). Soil pH especially alkalinity, low organic carbon, high carbonates (calcareous) and low redox potential of wetland primarily limit the Zn availability for rice. However, agricultural intensification, imbalanced nutrients and neglected micronutrient application further worsen the Zn-deficiency problem in rice. Indeed, zinc deficiency in rice is becoming one of the public health problems through malnutrition in many rice-based food adopting countries of Asia (Cakmak, 2008).

For rice, soil and foliar application of highly soluble zinc sulfate (ZnSO_4_) is a common Zn-fertilization to correct the Zn deficiency. However, the applied Zn got precipitated as hydroxides, carbonates, phosphates and sulfides as dictated by physico-chemical properties of the soil and resulted with very low fertilizer use efficiency (1–5%). Alternatively, exploring the soil bacteria, capable of solubilizing inorganic Zn and thereby increasing the availability for crop assimilation, is a viable option to achieve the objective of correcting the Zn deficiency and thereby overcoming the zinc malnutrition in human (He et al., 2010; Mäder et al., 2010). Several zinc solubilizing bacteria (ZSB) were characterized from tropical and temperate soils to provide plant available Zn (Hafeez et al., 2013). For example, *Gluconacetobacter* from sugarcane, *Bacillus* and *Pseudomonas* from soybean, rice and wheat capable of solubilizing zinc compounds such as oxide, carbonate, and phosphate were reported earlier (Saravanan et al., 2011). These ZSB strains produce variety of low molecular weight organic acids, particularly gluconic acid, dissolute the insoluble Zn; reduce the pH of the soil solution and thereby increase the plant available zinc (Hafeez et al., 2013). Inoculation of these bacteria enhanced the Zn uptake of rice (Vaid et al., 2014), maize (Goteti et al., 2013), wheat (Rana et al., 2012), green gram (Sharma et al., 2012), and soybean (Ramesh et al., 2014). Few studies also confirmed the ability of ZSB for biofortification in rice (Vaid et al., 2014) and wheat (Ramesh et al., 2014). However, their full potential to mitigate the zinc deficiency and to increase the grain-Zn is not yet explored due to poor understanding of microbe-soil-plant interactions.

The soil available zinc (Zn^2+^) is taken up by root membrane transport mechanisms in rice which include phytosiderophores (Bashir et al., 2010) and Zn-regulated transporters and iron (Fe)-regulated transporter-like protein (ZIP) family (Guerinot, 2000). In rice, several ZIPs including OsIRT1, OsIRT2, OsZIP1, OsZIP3, OsZIP4, OsZIP5, OsZIP7, and OsZIP8 were reported to be responsible for Zn uptake from soil, translocation within root and from root to shoot as well as for storage in grains (Ramesh et al., 2003; Ishimaru et al., 2005, 2006; Yang et al., 2009; Lee et al., 2010a,b). OsITR1 and OsITR2 are responsible for transport of Fe^2+^ from rhizosphere to root with less affinity to Zn (Ishimaru et al., 2006). OsZIP1, OsZIP3, OsZIP4, OsZIP5, and OsZIP8 are rice plasma membrane Zn transporters and are induced by Zn deficiency (Ramesh et al., 2003; Ishimaru et al., 2005; Yang et al., 2009; Lee et al., 2010a; Suzuki et al., 2012). The expression of most of the well-studied rice ZIP genes (*OsZIP1, OsZIP4, OsZIP5*) was controlled by the availability of divalent cations such as Zn^2+^, Fe^2+^, Cu^2+^, Mn^2+^ (Bughio et al., 2002; Ishimaru et al., 2005; Lee et al., 2010a). Few studies also confirmed that these transporter genes’ expression varied between root and shoot tissues of rice (Ishimaru et al., 2011). Similarly, Chen et al. (2008) reported the differential expression pattern of ZIP genes (*OsZIP1, OsZIP3*, and *OsZIP4*) between Zn-efficient and Zn-inefficient cultivars of rice. These ZIP genes varied their expression levels at different growth stages of rice from germination to grain filling (Ishimaru et al., 2011). The plant growth promoting rhizobacteria (PGPR) upon colonizing the roots, acidify the rhizosphere through organic acids and produce siderophores which facilitate the trace elements’ uptake by the crop plants. However, no attempts were made so far to elucidate the role of these zinc solubilizing PGPR strains to regulate the expression of metal transporter genes in the root. Understanding the interaction between rice plant and Zn solubilizing PGPR in terms of Zn transporter genes’ expression would help to alleviate the Zn deficiency as well as to improve the Zn fortification. In the present work, we have reported the root and shoot ZIP genes’ expression pattern of rice seedlings upon inoculating with a potential ZSB (*Enterobacter cloacae* strain ZSB14) under controlled condition. Our results suggest that the ZSB in rhizosphere of rice roots may regulate ZIP genes’ expression either directly or indirectly through Zn availability.

## Materials and Methods

### Bacterial Strain and Culture Condition

*Enterobacter cloacae* strain ZSB14, isolated and characterized from rhizosphere of rice, capable of solubilizing insoluble Zn compounds viz., ZnO (24.05 μg/ml of soluble Zn), ZnCO_3_ (19.37 μg/ml) and Zn_3_(PO_4_)_2_ (6.06 μg/ml) was used for this study. In order to maintain the Zn solubilizing potential of the strain, The culturing was routinely done in Bunt and Rovira medium containing 0.1% ZnO with and without agar (1.5%; Bunt and Rovira, 1955) at 30°C in an incubator (Lab Companion, USA).

### Rice Culture and ZSB Inoculation

Rice (*Orzya sativa*) cultivar Co51 of Tamil Nadu Agricultural University, Coimbatore was used for this experiment. De-husked healthy seeds were surface sterilized with sodium hypochlorite with 5% available chlorine for 10 min followed by five washes with sterile distilled water. The seeds were soaked in sterile distilled water for over-night for sprouting. Uniformly sprouted seeds were placed (10 seeds per plate) on Fe sufficient (Fe^+^) modified Hoagland medium (5 mM KNO_3_, 2 mM MgSO_4_, 2 mM Ca(NO_3_)_2_, 2.5 mM KH_2_PO_4_, 70 μM H_3_BO_3_, 1 μM MnCl_2_, 0.5 μM CuSO_4_, 10 μM NaCl, 0.2 μM Na_2_MoO_4_, 50 μM FeEDTA, 1.0 g/l MES buffer pH 5.8; 40 mM Sucrose; 8.0 g/l plant agar). For Fe-deficient (Fe^-^) condition, modified Hoagland medium lacking FeEDTA was used. Both under Fe^+^ and Fe^-^, five treatments for Zn nutrition were adopted viz., (i) no-zinc control; (ii) soluble Zn as ZnSO_4_ (5 μM); (iii) sparingly soluble ZnO (10 μM); (iv) ZnO with ZSB inoculation; (v) ZSB inoculation alone. ZnSO_4_ (5 μM) or ZnO (10 μM) was supplemented directly in modified Hoagland medium depending upon the treatment and seeds were placed. The plants were grown in a growth chamber with 12 h light (200 mole/m^2^/s) at 28°C. After 7 days of growth, the rice seedlings were inoculated with ZSB strain depending upon the treatment. For this, the strain ZSB14 was cultivated in Bunt and Rovira medium added with ZnO to achieve a final Zn concentration of 0.1% at 30°C till reached a final concentration of approximately 10^11^ colony forming units (cfu) per ml. The bacteria were pelletized by centrifugation at 5000 *g* for 20 min at room temperature and cell pellets were re-suspended in 10 mM MgSO_4_ and centrifuged. This operation was repeated and afterward the cell pellets were re-suspended in 10 mM MgSO_4_. The bacterial titer was adjusted to the OD_600_ of 0.05 (10^8^ cfu per ml) and 20 μL of bacterial suspension was then applied on each root of 7-days-old seedlings, right below the hypocotyl. After additional 7 days of incubation, the seedlings were removed carefully from the plates and assessed for ZIP gene expression.

### RNA Preparation and Real-Time RT-PCR Analysis

Total RNA from shoot and root of rice was extracted separately by following the procedure of Oñate-Sánchez and Vicente-Carbajosa (2008). The residual genomic DNA in the RNA preparation was digested with RNAse-free DNase I (New England Biolabs) until no amplicons were obtained when using RNA preparations directly in the PCR reaction with the primers for the actin gene (*OsACT1*). The primer details are provided in **Table 1**. Subsequently, complementary DNA (cDNA) was synthesized from 3 μg of DNA-free total RNA using Revert Aid H minus reverse transcriptase (Thermo Scientific) by primering with oligo d(T)_18_ (Invitrogen) in a 40-μL reaction mixture according to the manufacturer’s instruction. Real-time PCR was performed in Roche Lightcycler 480II (Roche, Switzerland) to quantify the transcripts of *OsZIP1, OsZIP4*, and *OsZIP5* (Primer details in **Table 1**) using SYBR Green (SYBR Premix ExTaq, Tli RNase H Plus, Takara) as the detection system. The constitutively expressed *OsACTIN1* gene was amplified as the reference gene. Changes in expression were calculated by relative quantification (ΔΔCt) method (Livak and Schmittgen, 2001) using threshold cycle (Ct) values of target and reference genes. For all real-time RT-PCR analyses, three biological replicates and two technical replicates were used. The size and intensity of amplified fragments were confirmed by gel electrophoresis.

**Table 1:** Primers used for quantitative real-time reverse transcription PCR.

Target gene	Primer name	Sequence	Reference
*OsZIP1*	OsZIP1-F	5′-CTCTTCAAGTTCCTCGCCGTCCT-3′	Ishimaru et al. (2005)
	OsZIP1-R	5′-CGGCCACGATTAATGAATGGGGTG-3′	
*OsZIP4*	OsZIP4-F	5′-GCGAAAGCAACAGTGATCATGGCGACTTTC-3′	Ishimaru et al. (2005)
	OsZIP4-R	5′-GCAGCTCTTGGTTGCTCTGAAGATCTCATG-3′	
*OsZIP5*	OsZIP5-F	5′-CTGGAGCTGGGAGTGGTGGT-3′	Lee et al. (2010a)
	OsZIP5-R	5′-ATGTCGACGAGCGCCATGTA-3′	
*OsACTIN1*	OsAct1-F	5′-GTATCCATGAGACTACATACAACT-3′	Lee et al. (2010a)
	OsAct1-R	5′-TACTCAGCCTTGGCAATCCACA-3′	

### Determination of Zn Content in Rice Plant

Rice seedlings washed until free from agar medium were oven-dried at 70°C for 5 h and digested with 15 ml triple acid mixture (nitric, sulfuric, and perchloric acid in the ratio of 9:2:1) for overnight. The volume of cooled digest was made up to 25 ml using deionized double distilled water and the dilutions were used for Zn estimation using atomic absorption spectrophotometer (GBS Scientific, Australia) at the wavelength of 213.86 nm.

### Statistical Analyses

All the data were subjected to statistical analysis with software, Microsoft Excel for Windows 2007 add-in with XLSTAT Version 2010.5.05 (XLSTAT, 2010). Statistically significant differences between the treatments were analyzed using analysis of variance (ANOVA) and Duncan’s Multiple Range Test (DMRT) at 5% significance level.

## Results

### *OsZIP1* Expression

**FIGURE 1:**
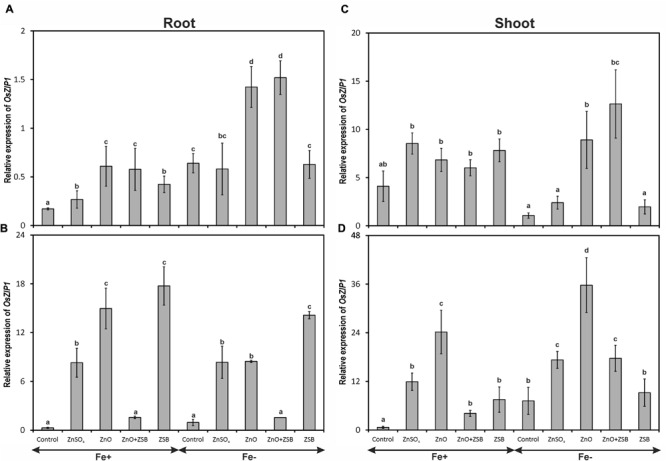
**Expression pattern of *OsZIP1* in root **(A,B)** and shoot **(C,D)** of rice seedlings as influenced by Fe and Zn. (A,C)** 7th day expression levels; **(B,D)** 14th day expression levels. Fe^+^, Fe sufficient condition; Fe^-^, Fe deficient condition. Control, No-zinc control; ZnSO_4_ at 5 mM; ZnO at 10 mM; ZSB, Zinc solubilizing bacteria (*Enterobacter cloacae* strain ZSB14) inoculation on 7th day. Relative mRNA abundance of *OsZIP1* was quantified and normalized with *OsACTIN1* gene on 7th day and 14th day. Data from real-time RT-PCR experiments were analyzed according to the 2^-ΔΔCt^ method. Means of six replicate values plotted, errors bars indicate the standard error. Values followed by the same letter in each panel are not significantly different from each other as determined by DMRT (*p* ≤ 0.05).

Both the Fe-levels and Zn-treatments significantly influenced the expression of *OsZIP1* (**Figure 1**). The 7-days-old Fe^-^ rice roots had higher copies of *OsZIP1* transcripts than those of Fe^+^ seedlings. Under Fe^-^ condition, ZnO addition recorded highest *OsZIP1* transcripts followed by no-Zn control. Under Fe^+^ condition, no-Zn control recorded lowest *OsZIP1* transcripts followed by ZnSO_4_ while, ZnO recorded the maximum relative expression among the treatments. Addition of ZnO in the medium up-regulated *OsZIP1* expression both in Fe^-^ and Fe^+^ conditions (**Figure 1A**). When ZSB-inoculated seedlings were assayed for *OsZIP1* expression after 7 days (14 days-old seedlings), the expression pattern of root *OsZIP1* was different than the earlier (**Figure 1B**). The ZSB inoculation substantially reduced the *OsZIP1* expression both in Fe-deficient and Fe-sufficient rice roots in the presence of ZnO. When ZnO was not in the medium, ZSB inoculated rice seedlings had maximum *OsZIP1* transcripts for both Fe-deficient and Fe-sufficient conditions. Under Fe^+^ condition, ZnSO_4_ amendment recorded higher *OsZIP1* transcripts than No-Zn and ZnO + ZSB, but lower than ZnO and ZSB. Under Fe^-^ condition, the same trend was noticed with the exception of ZnSO_4_ and ZnO with at par levels.

The *OsZIP1* levels of shoot were nearly 10-fold higher than root in the 7-days-old seedlings before exposure to ZSB inoculation (**Figure 1C**). However, the Fe-sufficient shoots did not show any significant difference within the Zn-treatments for the level of *OsZIP1* transcripts, while the Fe-deficient shoots showed significant difference between them. The no-Zn controls and ZnSO_4_ had lowest shoot *OsZIP1* while ZnO amended Fe^-^ rice recorded maximum expression. After 7-days of ZSB inoculation, *OsZIP1* levels had significant different in the Zn-treatments. The ZSB inoculation considerably reduced the *OsZIP1* expression of the rice shoot (**Figure 1D**). When comparing the ZnO and ZnO + ZSB, nearly 50% reduction in *OsZIP1* expression was recorded due to ZSB inoculation in both Fe^-^ and Fe^+^ conditions. In shoot also, the *OsZIP1* expression was reduced in the presence of Fe in the medium.

### *OsZIP4* Expression

**FIGURE 2:**
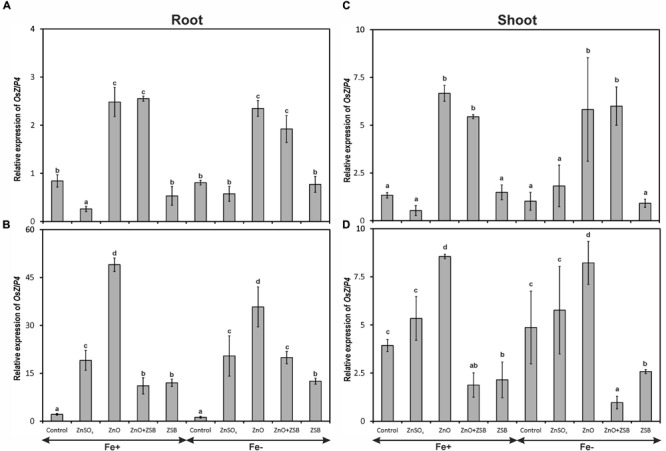
**Expression pattern of *OsZIP4* in root **(A,B)** and shoot **(C,D)** of rice seedlings as influenced by Fe and Zn. (A,C)** 7th day expression levels; **(B,D)** 14th day expression levels. Fe^+^, Fe sufficient condition; Fe^-^, Fe deficient condition. Control, No-zinc control; ZnSO_4_ at 5 mM; ZnO at 10 mM; ZSB, Zinc solubilizing bacteria (*Enterobacter cloacae* strain ZSB14) inoculation on 7th day. Relative mRNA abundance of *OsZIP4* was quantified and normalized with *OsACTIN1* gene on 7th day and 14th day. Data from real-time RT-PCR experiments were analyzed according to the 2^-ΔΔCt^ method. Means of six replicate values plotted, errors bars indicate the standard error. Values followed by the same letter in each panel are not significantly different from each other as determined by DMRT (*p* ≤ 0.05).

The expression of *OsZIP4* gene was significantly influenced by different Zn-treatments but not by Fe^-^ levels (**Figure 2**). Both Fe^+^ and Fe^-^ rice responded similar pattern of *OsZIP4* expression in the root and shoot of rice seedlings. The root of 7-days-old rice seedlings recorded significantly highest *OsZIP4* transcripts due to ZnO followed by no-Zn control under Fe-deficient and sufficient conditions (**Figure 2A**). The ZnSO_4_ amendment down-regulated the *OsZIP4* significantly than other treatments. When the ZSB strain was inoculated, the root *OsZIP4* showed remarkable difference of expression after 7 days. Irrespective of treatments, the level of relative expression of *OsZIP4* at 14 days-old seedling had been increased nearly 10-fold than 7 days-old plants (**Figure 2B**). Among the rice seedlings exposed to different amendments, ZnO addition significantly increased the root-*OsZIP4* transcripts followed by ZnSO_4_, while the ZnO + ZSB and ZSB alone significantly reduced the expression. The no-Zn controls did not show any variations in their root *OsZIP4* transcript levels between two observations.

With reference to shoot *OsZIP4*, the level of expression remained same between Fe^+^ and Fe^-^ seedlings after 14 days of incubation (**Figures 2C,D**). The shoot of 7-days-old rice seedlings before ZSB inoculation exposed to ZnO had significantly higher *OsZIP4* transcripts that ZnSO_4_ and no-Zn controls of both Fe-deficient and sufficient rice plants (**Figure 2C**). When ZSB was inoculated on 7th day, the *OsZIP4* significantly reduced in ZnO + ZSB treatment to a tune of 77 and 88% for Fe-sufficient and deficient rice plants, respectively as compared to ZnO treatment (**Figure 2D**). Irrespective to Fe-levels, ZnO amended uninoculated plants remained constant level of expression for both the assessments; whereas ZnSO_4_ amended plants increased their *OsZIP4* expression levels to ninefold after 7 days of additional incubation.

### *OsZIP5* Expression

**FIGURE 3:**
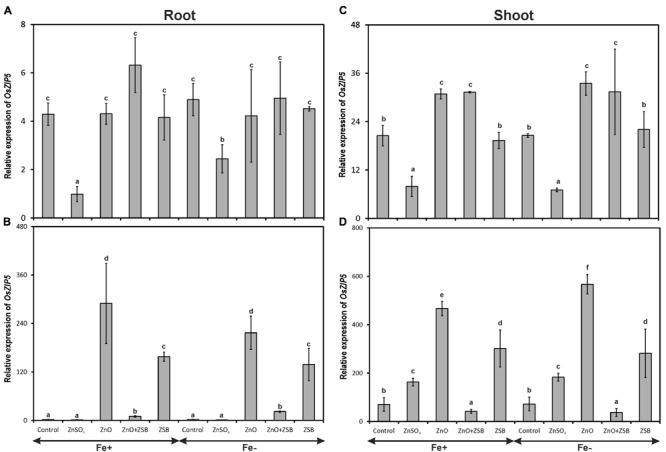
**Expression pattern of *OsZIP5* in root **(A,B)** and shoot **(C,D)** of rice seedlings as influenced by Fe and Zn. (A,C)** 7th day expression levels; **(B,D)** 14th day expression levels. Fe^+^, Fe sufficient condition; Fe^-^, Fe deficient condition. Control, No-zinc control; ZnSO_4_ at 5 mM; ZnO at 10 mM; ZSB, Zinc solubilizing bacteria (*Enterobacter cloacae* strain ZSB14) inoculation on 7th day. Relative mRNA abundance of *OsZIP5* was quantified and normalized with *OsACTIN1* gene on 7th day and 14th day. Data from real-time RT-PCR experiments were analyzed according to the 2^-ΔΔCt^ method. Means of six replicate values plotted, errors bars indicate the standard error. Values followed by the same letter in each panel are not significantly different from each other as determined by DMRT (*p* ≤ 0.05).

The transcripts of *OsZIP5* were strongly found in shoots and weakly in roots of 7-days-old rice seedlings (**Figure 3**). Like *OsZIP4, OsZIP5* also did not show significant response to Fe levels. In the roots of 7-days-old rice seedlings, the no-Zn and ZnO amended rice seedlings showed significantly higher levels of *OsZIP5* in both Fe-sufficient and deficient conditions (**Figure 3A**). The ZnSO_4_ amended rice plants had the least expression of *OsZIP5* in their roots. After ZSB inoculation and 7 days incubation, the pattern of *OsZIP5* expression was different than those of before inoculation. After additional 7 days of incubation, the rice seedlings exposed to ZnO alone had nearly 60-fold increased *OsZIP5* transcripts in both Fe^+^ and Fe^-^ conditions (**Figure 3B**). However, ZSB inoculation alone also induced *OsZIP5* in Fe^+^ and Fe^-^ rice roots but significantly lower than ZnO amendment. The ZnO + ZSB inoculation, ZnSO_4_ and no-Zn controls had least *OsZIP5* transcripts in their Fe^+^ and Fe^-^ roots.

The response of rice shoot *OsZIP5* was similar to that of root but with twofold increased levels than roots (**Figures 3C,D**). There was no significant difference between Fe^+^ and Fe^-^ plants in terms of shoot *OsZIP5* expression. The ZnSO_4_ amendment in the medium down-regulated the *OsZIP4* in Fe^+^ and Fe^-^ shoots, whereas no-Zn controls and ZnO amendments had significantly higher copies of *OsZIP5* transcripts (**Figure 3C**). When ZSB was inoculated to their respective treatment plants, there was significant effect found due to ZSB inoculation. ZnO + ZSB inoculation significantly down-regulated the *OsZIP5* to a tune of 91 and 95% for Fe^+^ and Fe^-^ plants, respectively as compared to ZnO amended rice shoots (**Figure 3D**). The ZSB inoculation without ZnO up-regulated the *OsZIP5* expression in shoots after 7-days of incubation. The no-Zn and ZnSO_4_ also had significantly higher levels of *OsZIP5* transcripts than ZnO + ZSB treatment.

### Leave Chlorosis of Rice Seedlings

**FIGURE 4:**
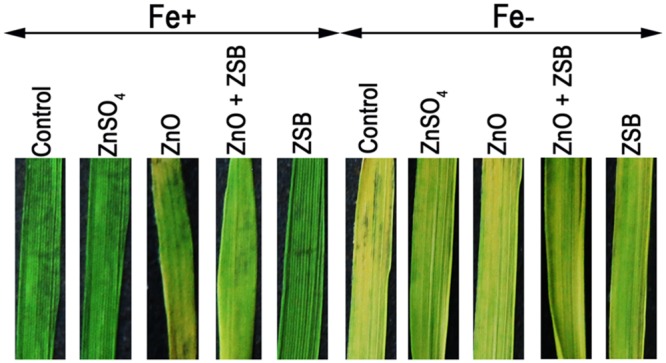
**Leaf color of 14-days-old rice seedlings after exposure to different Zn treatments under Fe^+^ and Fe^-^ conditions.** Control, No-zinc control; ZnSO_4_ at 5 mM; ZnO at 10 mM; ZSB, Zinc solubilizing bacteria (*Enterobacter cloacae* strain ZSB14) inoculation on 7th day.

We examined the role of Fe and ZSB-bound Zn availability on metal uptake of rice (Fe and Zn) in terms of chlorosis of leaves. The color intensity of rice leaves after 14-days of exposure to various Zn treatments under Fe^+^ and Fe^-^ conditions showed significant difference (**Figure 4**). The Fe^+^ condition made rice leaves with dark intensity while the Fe^-^ showed chlorosis. Under Fe^+^ condition, ZnO induced the chlorosis of leaves, while with ZSB inoculation, the chlorosis was reduced. The ZnSO_4_ and ZSB alone did not show any chlorosis at all. Under Fe^-^ condition, no-Zn and ZnO showed severe yellowing, while the ZnSO_4_ and ZSB had less chlorosis.

### Zn Content of Rice Seedlings

The root and shoot Zn content of rice seedlings before ZSB inoculation (7th day) was significantly influenced by Zn amendments and also due to Fe conditions. In 7-days-old seedlings, ZnSO_4_ recorded 198 and 280% higher root Zn and 108 and 121% higher shoot Zn than Fe^+^ and Fe^-^ no-Zn controls, respectively (**Table 2**). ZnO also increased the root and shoot Zn of rice seedlings than no-Zn controls, which were trivial (14–18%) as compared to ZnSO_4_. The Zn uptake measured as zinc content of rice seedlings after ZSB inoculation (14th day) was also significantly influenced by Zn sources. Fe^-^ condition increased the Zn contents of root and shoot significantly than Fe^+^ for Zn treatments (ZnSO_4_ and ZnO + ZSB) but not for no-Zn and ZSB alone controls at 14th day (**Table 2**). Among the various treatments enforced, the ZnSO_4_ recorded maximum root and shoot Zn contents (198.25 and 154.26 mg/g for Fe^-^ and 145.15 and 125.37 mg/g for Fe^+^ respectively). The ZnO + ZSB treated plants recorded 157.46 and 124.13 mg/g of Zn in root and shoot, respectively under Fe-deficient condition, and 87.24 and 90.16 mg/g for Fe-sufficient conditions. The ZnO alone treated plants had very little increase of Zn content as compared to no-Zn control. ZSB inoculation alone had at par root and shoot Zn levels as that of no-Zn controls for Fe^+^ and Fe^-^ rice seedlings.

**Table 2:** Effect of different Zn sources and ZSB inoculation on the Zn content of rice seedlings (Cultivar Co51).

Treatments	Zinc content (mg/g dry weight)			
	7th day		14th day	
	Root	Shoot	Root	Shoot
**Fe (+)**				
Control	41.7 (±2.0)^a^	56.7 (±2.3)^a^	52.4 (±3.0)^a^	75.5 (±3.3)^a^
ZnSO_4_	127.2 (±4.4)^c^	118.4 (±5.3)^c^	145.2 (±2.2)^d^	125.4 (±5.1)^e^
ZnO	46.7 (±1.2)^b^	66.2 (±1.6)^b^	67.2 (±1.0)^b^	80.2 (±2.4)^b^
ZnO + ZSB	47.4 (±1.3)^b^	66.7 (±2.3)^b^	87.2 (±3.1)^c^	90.2 (±1.3)^c^
ZSB	42.1 (±2.4)^a^	55.7 (±1.1)^a^	51.5 (±0.6)^a^	70.2 (±1.2)^a^
**Fe (-)**				
Control	42.6 (±1.0)^a^	58.2 (±2.0)^a^	58.2 (±1.2)^a^	76.2 (±1.6)^a^
ZnSO_4_	158.5 (±5.3)^d^	128.8 (±6.3)^c^	198.3 (±8.1)^e^	154.2 (±3.7)^f^
ZnO	48.5 (±2.1)^b^	67.5 (±1.3)^b^	66.3 (±5.5)^b^	108.5 (±2.2)^d^
ZnO + ZSB	48.4 (±1.2)^b^	68.7 (±2.4)^b^	157.5 (±2.2)^d^	124.1 (±2.4)^e^
ZSB	42.8 (±2.3)^a^	57.3 (±1.2)^a^	61.2 (±3.1)^ab^	71.5 (±3.6)^a^

*Values are mean (± standard error; *n* = 3) and values followed by the same letter in each column are not significantly different from each other as determined by DMRT (*p* ≤ 0.05). Fe (+), Fe sufficient condition; Fe (-), Fe deficient condition; Control, No-zinc control; ZSB, Zinc solubilizing bacteria (*Enterobacter cloacae* strain ZSB14) inoculation on 7th day.*

## Discussion

Exploiting the ZSB for alleviating Zn-deficiency as well as for Zn-fortification in food grains like rice could be a promising agronomical approach to minimize the Zn-deficiency in human being. Keeping in view the unambiguous benefits of ZSB (Hafeez et al., 2013), through the present investigation, we reported that ZSB inoculation to rice could alter the expression of zinc transporting genes of rice based on the Zn solubilization and thereby regulate the uptake of zinc.

The ZIP family transporters are well-characterized and are suggested to be the primary uptake system for Zn in plants (Guerinot, 2000; Mäser et al., 2001). Most of these ZIP genes are induced by Zn deficiency (Ramesh et al., 2003; Ishimaru et al., 2005; Chen et al., 2008) and their expression pattern varied between root and shoot system. *OsZIP1* was shown to be expressed higher levels in roots than shoots under Zn-deficient condition (Ramesh et al., 2003; Ishimaru et al., 2005). Chen et al. (2008) observed that *OsZIP1* was up-regulated in Zn-deficient roots, but no visible transcripts detected in shoots of both Zn-efficient and Zn-inefficient rice genotypes. In contrast to these, Ramegowda et al. (2013) found that *OsZIP1* over-expressing transgenic finger millet showed higher expression of *OsZIP1* in leaves under Zn-sufficient condition. In the present work also, we found higher expression of *OsZIP1* in shoot than root in 7-days-old rice and the *OsZIP1* expression was influenced by Fe availability apart from zinc. The rice grown for 7-days under Fe-sufficient condition had relatively lower *OsZIP1* transcripts than those plants grown in Fe-deficient condition. Among the two Zn-treatments, sparingly soluble ZnO up-regulated the *OsZIP1* as compared to highly soluble ZnSO_4_ before ZSB inoculation. This is in accordance with the earlier findings that the zinc abundance reduced the root *OsZIP1* expression (Ramesh et al., 2003; Ishimaru et al., 2005). However, in the present work, when the rice plants grown with ZnO had highest *OsZIP1* expression in their roots after 7 days which was higher than no-zinc control. This implies that the sparingly soluble ZnO could not supply the available Zn in the growth medium of rice and subsequently cause more stress than no-Zn condition. Further investigations are needed to understand how the ZnO induced the ZIP transporters higher than no-Zn condition. However, when ZSB was inoculated on 7-days-old rice seedling, considerable reduction in *OsZIP1* expression was noticed in both Fe^+^ and Fe^-^ root and shoot of rice. This might be due to the ZSB-mediated Zn solubility and availability in the medium as well as the ZSB-mediated rhizospheric effects. Interestingly, ZSB inoculation increased the root and shoot *OsZIP1* expression even in the absence of Zn.

In the present work, Fe^+^ and Fe^-^ conditions did not alter the expressions of *OsZIP4* and *OsZIP5* as that of *OsZIP1* which is in accordance with the earlier works (Ishimaru et al., 2005, 2007; Lee et al., 2010a). *OsZIP1* is primarily associated with metal uptake from rhizosphere (Ramesh et al., 2003), while *OsZIP4* and *OsZIP5* are involved in the translocation of Zn with in the plant (Ishimaru et al., 2005) might be the reason, why these genes are not regulated due to Fe levels. The previous works confirmed that *OsZIP4* in Zn-deficient rice was expressed in meristem and vascular bundles of roots and shoots and is responsible for Zn translocation to various plant parts that require Zn (Ishimaru et al., 2011). As the transporters involving in metal uptake from soil may have non-specific uptake of the ions such as Zn, Fe, Cu, Cd, Mn from soil to the root, these genes’ expression was regulated based upon the affinity of the metals. However, the transporters responsible for translocation of metals within the plant had less impact of other metal species. For example, the transporters *OsZIP4, OsZIP5*, and *OzZIP8* responsible for Zn translocation in rice are not influenced by Fe^+^, while the *OsZIP1* and *OsITR1* responsible for Zn and Fe uptake from soil respectively, were also influenced by other metals (Lee and An, 2009). The present results are in accordance with these findings. In the present work, ZnSO_4_ in the growth medium made Zn sufficient condition and thereby reduced the *OsZIP4* expressions in both root and shoot. When ZnO was amended, the relative *OsZIP4* expression was significantly higher than no-Zn control which means that the addition of ZnO cause more stress to the rice than no-Zn. When the ZSB was inoculated on 7th day and incubated for additional 7-days, the relative expression of *OsZIP4* got varied in those treatments which imply that rhizosphere colonization of ZSB either directly or indirectly regulates *ZIP* genes of rice. Compare to ZnO treatment, ZnO + ZSB reduced the *OsZIP4* expression revealed that the ZSB-mediated solubilization of ZnO enhanced the uptake of Zn and thereby reduced the Zn deficiency. The down-regulation of *OsZIP4* found in rice shoot due to ZSB inoculation implies that the ZSB-bound Zn release has been effectively translocated to the shoot system also. Compare to no-Zn control, ZSB inoculation in the absence of ZnO up-regulated root *OsZIP4* but down-regulated the shoot *OsZIP4*. However, compare to *OsZIP1*, the ZSB-mediated regulation of *OsZIP4* was relatively low. Hence, further investigation is needed to understand this variation between ZIP transporters’ response for ZSB inoculation.

*OsZIP5* is a plasma membrane-bound transporter responsible for Zn translocation within the rice plant. Expression of *OsZIP5* is mainly regulated by Zn levels and Zn deficient condition up-regulated the expressions in both shoot and root (Lee et al., 2010a). Over-expression of *OsZIP5* over-expressed plants showed sensitive to excess Zn, while the *OsZIP5* knock-out plants had high Zn tolerance (Ishimaru et al., 2011). In the present experiment, the expression pattern of *OsZIP5* was differed from *OsZIP1* and *OsZIP4* in several treatments. Before ZSB inoculation, no-Zn and ZnO applied rice plants, which are suffered with Zn deficiency had maximum *OsZIP5* expression both in root and shoot. ZnSO_4_ in the medium down regulated the expression of root and shoot *OsZIP5*. When ZSB was inoculated to ZnO and no-Zn plants, *OsZIP5* was in low copies in ZnO amended plants, while No-Zn but ZSB inoculated plants had significantly higher transcripts. This result implies that ZSB had significant influence on *OsZIP5* by providing soluble Zn from ZnO while in the absence of Zn, ZSB up-regulated *OsZIP5* as that of *OsZIP1*. Hence, it is clear from these experiments that ZSB had direct impact on *OsZIP1* and *OsZIP5* and for *OsZIP4*, the regulation is dependent of Zn-availability due to the functioning of ZSB.

Several previous studies also confirmed that ZSB inoculation enhanced the exchangeable Zn in the soil or rhizosphere of crops through organic acid production and enhanced microbial processes and subsequently improved the Zn uptake (Oburger et al., 2009; Ramesh et al., 2014; Shakeel et al., 2015). As supportive to these findings, in the present work, the Zn content of shoot and root of ZnO + ZSB inoculated rice plants was higher than no-Zn, ZnO alone and ZSB alone plants, but lower than ZnSO_4_ amended plants. This was further evident from the observation on the chlorosis of rice leaves in the present experiment (**Figure 4**). Fe and Zn sufficient conditions did not show any chlorosis, while ZnO induced the chlorosis implies that ZnO may affect the Fe uptake along with Zn. Hence, inoculation of ZSB in the root zone improved the Zn uptake and translocation within the plant and thereby increased the Zn contents of root and shoot compared to no-Zn and ZnO alone controls.

## Conclusion

In the present investigation, we proved that the inoculation of ZSB under controlled condition can able to regulate some of the Zn-regulated transporters family genes and thereby controlled the Zn uptake in rice seedlings. Zn sufficient condition created by ZnSO_4_ down regulated *OsZIP4* and *OsZIP5* both in root and shoot of rice. The application of sparingly soluble ZnO as Zn source created severe Zn related stress to the rice, which up-regulated all the ZIP genes. Upon inoculation of ZSB, the expression levels of *OsZIP1, OsZIP4*, and *OsZIP5* were reduced. In the absence of Zn source, ZSB inoculation could regulate *OsZIP1* and *OsZIP5* but not the *OsZIP4*. These results are evident that the ZSB inoculation as PGPR could regulate the Zn uptake and translocation in rice plant and thereby zinc fortification in rice grains.

## Author Contributions

The experiments were planned and executed together by SK and DB. SK undertook the data analysis. The interpretation of results and manuscript preparation were done by DB.

## Conflict of Interest Statement

The authors declare that the research was conducted in the absence of any commercial or financial relationships that could be construed as a potential conflict of interest.
